# Heparan Sulfate and Enoxaparin Interact at the Interface of the Spike Protein of HCoV-229E but Not with HCoV-OC43

**DOI:** 10.3390/v15030663

**Published:** 2023-03-01

**Authors:** Virginia Fuochi, Giuseppe Floresta, Rosalia Emma, Vincenzo Patamia, Massimo Caruso, Chiara Zagni, Federica Ronchi, Celestino Ronchi, Filippo Drago, Antonio Rescifina, Pio Maria Furneri

**Affiliations:** 1 Department of Biomedical and Biotechnological Sciences (Biometec), University of Catania, 95124 Catania, Italy; 2 Center of Excellence for the Acceleration of Harm Reduction (Coehar), University of Catania, 95124 Catania, Italy; 3 Department of Drug and Health Sciences (DSFS), University of Catania, 95125 Catania, Italy; 4 Delim Cosmetics & Pharma S.R.L., 20090 Vimodrone, MI, Italy

**Keywords:** heparan sulfate, enoxaparin, coronavirus, HCoV-229E, HCoV-OC43, APN, 9-*O*-Ac-Sia, molecular docking

## Abstract

It is known that the spike protein of human coronaviruses can bind to a secondary receptor, or coreceptor, to facilitate the virus entry. While HCoV-229E uses human aminopeptidase N (hAPN) as a receptor, HCoV-OC43 binds to 9-*O*-acetyl-sialic acid (9-*O*-Ac-Sia), which is linked in a terminal way to the oligosaccharides that decorate glycoproteins and gangliosides on the surface of the host cell. Thus, evaluating the possible inhibitory activity of heparan sulfate, a linear polysaccharide found in animal tissues, and enoxaparin sodium on these viral strains can be considered attractive. Therefore, our study also aims to evaluate these molecules’ antiviral activity as possible adsorption inhibitors against non-SARS-CoV. Once the molecules’ activity was verified in in vitro experiments, the binding was studied by molecular docking and molecular dynamic simulations confirming the interactions at the interface of the spike proteins.

## 1. Introduction

Coronaviruses’ history begins in the 1930s [1,2]. However, in the last 60 years, there has been a particular interest in their relationship with human diseases, thanks to the discovery of the first two human strains, OC43 [3,4] and 229E [5]. Subsequently, this interest became more and more consolidated with the discovery of the so-called pathogenic coronaviruses [6,7,8,9] and, more appropriately, with the appearance of the pandemic viruses SARS-CoV-1 [10], MERS-CoV [11], and SARS-CoV-2 [12].

The latest pandemic caused by SARS-CoV-2 has enormously raised the crucial role of pericapsid glycoproteins in the pathogenesis of the infection [13,14], in the secondary immune response [15], for vaccine [16], and also in the role that this protein could have in controlling the T-cell-mediated response [17].

The genomic organization of human coronavirus has shown a variability among structural proteins between α-coronavirus and β-coronavirus and the protein involved in cell interactions [13,14,18,19].

It is known that the spike protein of human coronaviruses can bind to a secondary receptor or coreceptor to facilitate such entry. MERS-CoV uses sialic acid as a coreceptor and its main receptor, DPP4 [20]. Human CoV-NL63 uses ACE2 as a receptor and proteoglycans heparan sulfate (HS) as a coreceptor [21], while SARS-CoV-1 pseudovirus binds to HS as a coreceptor for infectivity. Differently, HCoV-229E uses human aminopeptidase N (hAPN) as a receptor [22], whereas HCoV-OC43 binds to 9-*O*-acetyl-sialic acid (9-*O*-Ac-Sia), which is linked in a terminal way to the oligosaccharides that decorate glycoproteins and gangliosides on the surface of the host cell [23].

Precisely, the attachment of the virus to the host cell is initiated by the interactions between the protein S and its receptor. The site of receptor binding domains (RBDs) within the S1 region of a coronavirus protein S varies for each coronavirus. For example, the spike proteins S of SARS-CoV-1 and SARS-CoV-2 attach the virus to its cellular receptor, angiotensin-converting enzyme 2 (ACE2) [24,25].

Nowadays, in silico medicinal chemistry has generated major interest in the research field, leading to significant results. In the last few years, our research group has gained experience in computational drug design [26,27,28,29,30,31,32,33,34,35,36] and identified several hit compounds as inhibitors of the fusion process of SARS-CoV-2 HR1 [37,38], as well as on artificial intelligence in de novo drug design for COVID-19 pharmaceutical research [35]. Moreover, there has been a renewed interest in the search for natural molecules with antiviral activity [38,39,40] and the potential antiviral efficacy of glycans and proteoglycans [41,42].

Proteoglycans are heavily glycosylated proteins and a major component of non-structural extracellular matrices. They are composed of independent structural domains, the sequences and arrangements of which are highly conserved and discretely glycosylated, thus determining a varying degree of matrices organization. Therefore, these molecules participate in maintaining the bulk, shape, and strength of tissues in vivo. Moreover, they are critically important for cell growth, survival, and differentiation [43,44,45,46]. Furthermore, recent evidences have shown their use in clinical practices as biomarkers in diagnosis in various pathologies [47,48,49,50,51,52].

Several published studies have suggested that numerous viruses use the heparan sulfate component of cell surface heparan sulfate proteoglycans (HSPGs), which are ubiquitously expressed, as an initial receptor to attach to cells [53,54]. Moreover, there are indications that the SARS-CoV-2 spike also interacts with HSs [55] and facilitates the attachment of spike-bearing viral particles to the cell surface to promote viral entry [56]. In fact, it has been demonstrated that the SARS-CoV-2 spike protein interacts with heparan sulfate and ACE2 through the RBD [57].

According to the above observations, there is a need to investigate the effects of heparan sulfate or enoxaparin sodium (EX) on virus adsorption and/or entry to develop any potential pharmaceutical formulation to be used in the prevention of coronavirus disease. Indeed, EX is a drug usually exploited in pulmonary disease treatment in place of heparin [58]. It is produced starting from standard heparin and belongs to the low-molecular-weight heparins (LMWH) group: a strongly acidic chain composed of the monosaccharides glucosamine and glucuronic acid linked by disulfide bridges [59]. Moreover, some studies showed that therapeutic-dose LMWH had safer and/or more significant effects than standard institutional heparin [60,61,62].

Therefore, our study aimed to develop a pilot study to evaluate the antiviral activity of both HS and EX and/or their possible adsorption-inhibiting activity by using HCoV-229E and HCoV-OC43. Once the molecules’ activity was verified in in vitro experiments, the binding ability was evaluated by molecular docking and molecular dynamic simulations.

## 2. Materials and Methods

### 2.1. Chemicals, Cellular Lines, and Viruses

Heparan sulfate (HS) and enoxaparin sodium (EX) were kindly supplied by Techdow Pharma S.r.l Assago Milanofiori (MI) Italy.; MRC5 (Lung Normal Fibroblast Cells ATCC^®^ CCL171™) and HCT-8 (Human Colon Adenocarcinoma Epithelial Cells HRT-18 CCL-244™) were purchased from American Type Culture Collection (ATCC, Manassas, VA, USA) and cultured as follows.

MRC5 cells were cultured in DMEM high glucose medium supplemented with 2 mM l-glutamine, 100 U/mL penicillin–streptomycin mixture, and 10% fetal bovine serum (FBS), at 37 °C, in a 5% CO_2_ humidified incubator. HCT-8 cells were cultured in RPMI-1640 medium supplemented with 2 mM l-glutamine, 100 U/mL penicillin–streptomycin mixture, and 10% of FBS, at 37 °C, in a 5% CO_2_ humidified incubator. Adherent sub-confluent cell monolayers were prepared in growth medium (2% FBS) in 96-well plates for cytotoxicity assays and viral inhibition tests.

HCoV-229E and HCoV-OC43 strains were obtained from ATCC, passaged, and amplified on MRC5 and HCT8 cells, respectively.

### 2.2. Cell Viability Assay by MTT

The evaluation of the cytotoxic effects of the solutions under examination on human pulmonary eukaryotic cells was performed employing the MTT assay as previously reported [63]. Therefore, cell viability was measured by colorimetric reduction of MTT enzymatically catalyzed by mitochondrial succinate dehydrogenase. The color intensity was measured at 570 nm using a spectrophotometric reader. Six assays for each sample were performed, and the results were expressed as mean ± SD.

### 2.3. Cell Viability Assay by Air-Liquid Interface (ALI) Exposure

The evaluation of the cytotoxic effects was also performed by ALI exposure. MRC5 cells were seeded in 12 mm Transwells^®^ inserts (Corning Incorporated, Corning, NY, USA) at a density of 1.75 × 10^5^ cells/mL sustained by 1 mL of medium in the basal compartment of each well and 0.5 mL in the apical compartment of each insert, 48 h before exposure. When the cells reached 80% confluency, the apical medium was removed from each insert. As previously described, two inserts per test product were transitioned to the exposure chamber with 20 mL of medium in the basal compartment to perform the ALI exposure [64]. ALI exposure was carried out using a Borgwaldt LM4E vaping machine with an aerosol nebulizer (Borgwaldt-Kc, Hamburg, Germany) attached to vaporize the solutions object of our study (Figure S1).

The cell exposure chambers used in this study were previously described by Azzopardi et al. [65,66]. (Figure S2) [65,66].

In order to evaluate the cytotoxicity of the aerosols generated by the substances on cells, the nebulizer was loaded with 2 mL of solutions, HS and EX, in two different concentrations: 10 mg/mL and 2.5 mg/mL. In addition, 2 mL of saline was vaporized as a negative control, and 2 mL of 30% DMSO solution as a positive control. Nebulization was performed with a regimen equal to a volume of 100 mL for 10 s at a frequency of 20 s interpuffs until completion of the solution, i.e., for a time of 15 min × 30 puffs.

### 2.4. Antiviral Activity

The in vitro evaluation of antiviral activity on coronavirus 229E and OC43 strains was performed in the adsorption phase on MRC5 and HCT-8 cells, respectively. Therefore, cells were exposed to the substance and simultaneously infected with the virus (MOI 0.01). Each assay provided different internal controls: K cells (cells not exposed to the virus or substance), K virus (cells infected with the virus but not exposed to the substance), and K substances (cells exposed to the substance but not infected with the virus).

The infectivity of viruses was determined by the MTT method: the reciprocals of viral dilution that resulted in a 50% reduction of absorbance of formazan in the infected cells at 48–72 h was determined as the MTT ID50 (50% infective dose). The antivirus assay was based on inhibiting virus-induced cytopathogenicity (CPE) in cells. Briefly, subconfluent monolayers grown in 96-well tissue culture plates were treated with or without various concentrations of HS and EX at doses below the CC_50._ Subsequently, the virus was added for the adsorption (2 h at 37 °C, 5% CO_2_), and then, cells were maintained in the incubator without a wash for 3–5 days to obtain a complete cytopathic effect. Finally, the viability of virus-infected cells was quantified by the the MTT method. Data of viral inhibition were calculated as the percentage of the CPE with respect to the negative control (K cells).

### 2.5. Statistical Analysis

All experiments were performed at least three times, and data were summarized using the mean (±SD). Where applicable, data were analyzed by one-way ANOVA with correction for multiple comparisons by Bonferroni. All results with a *p*-value < 0.01 were considered significant. All results and graphs were generated using GraphPad^®^ Prism ver. 8.4.2.

### 2.6. Molecular Modeling

The 2D chemical structures of the two studied molecules (PubChem CID: 70678539 and 60196282) were built using Marvin Sketch, and all the structures were subjected to molecular mechanics energy minimization using the MMFF94 force field present in the same software [67]. The 3D geometry of all compounds was then optimized using the PM3 Hamiltonian [68], as implemented in MOPAC 2016 package, assuming a pH of 7.0 [69]. Several versions of the heparan sulfate reported in the literature [70] were tested. No significant differences were obtained when working with different variants. The same portion of the molecule achieves the main interactions, and the side chains are pointing outside the binding cavities, hence not relevant for protein/heparan interactions. Molecular docking experiments were achieved with AutoDock 4.2.6, and AutoDock Vina provided in YASARA (v. 22.5.22, YASARA Biosciences GmbH, Vienna, Austria) [71,72] using the crystal structures of HCoV-229E RBD Class V in complex with human APN (PDB ID: 6U7G) and cryo-EM structures of coronavirus OC43 S glycoprotein trimer (PDB ID: 6NZK) [73] collected from the Protein Data Bank (PDB, http://www.rcsb.org/pdb, accessed on 10 September 2022) and the Lamarckian genetic algorithm (LGA). The proteins have been optimized using YASARA software. The maps were made by AutoGrid (4.2.6) with an architecture of 0.375 Å and an extension encompassing all atoms spanning 5 Å from the exterior of the structure of the ligand. Point charges were originally defined according to the AMBER03 force field and then damped to mimic the less polar Gasteiger charges used to optimize the AutoDock scoring function. All parameters were used at their default settings. In the docking tab, the macromolecule and ligand were selected, and LGA parameters were set as ga_cauchy_beta = 1.0, ga_mutation_rate = 0.02, ga_runs = 100, ga_crossover_mode = two points, ga_cauchy_alpha = 0.0, ga_pop_size = 150, ga_num_generations = 27,000, ga_crossover_rate = 0.8, ga_num_evals = 25,000,000, ga_elitism = 1, number of generations for picking worst individual = 10. The MD simulations of the complexes were performed with the YASARA structure package. A periodic simulation cell with boundaries extending 8 Å [74] from the surface of the complex was employed. The box was filled with water, with a maximum sum of all water bumps of 1.0 Å and a density of 0.997 g/mL.

The setup included optimizing the hydrogen bonding network [75] to increase the solute stability and a p*K*_a_ prediction to fine-tune the protonation states of protein residues at the chosen pH of 7.4 [76]. NaCl ions were added with a physiological concentration, neutralizing the cell. Water molecules were deleted to readjust the solvent density to 0.997 g/mL.

The simulation was run using the GAFF2 force fields [77] with AM1BCC [78] calculated charges for ligands, ff14SB force fields [79] for the solute, and TIP3P force fields for water. The cutoff was 10 Å for van der Waals forces (the default used by AMBER) [80], and no cutoff was applied to electrostatic forces (using the particle mesh Ewald algorithm) [81]. The equations of motions were integrated with multiple time steps of 2.5 fs for bonded interactions and 5.0 fs for nonbonded interactions at a temperature of 298 K and a pressure of 1 atm using algorithms described in detail previously [82,83]. A short MD simulation was run on the solvent only to remove clashes. The entire system was then energy-minimized using the steepest descent minimization to remove conformational stress, followed by a simulated annealing minimization until convergence (<0.01 kcal/mol Å). Finally, 100 ns MD simulations without any restrictions were conducted, and the conformations of each system were recorded every 200 ps.

## 3. Results and Discussion

**Figure 1:**
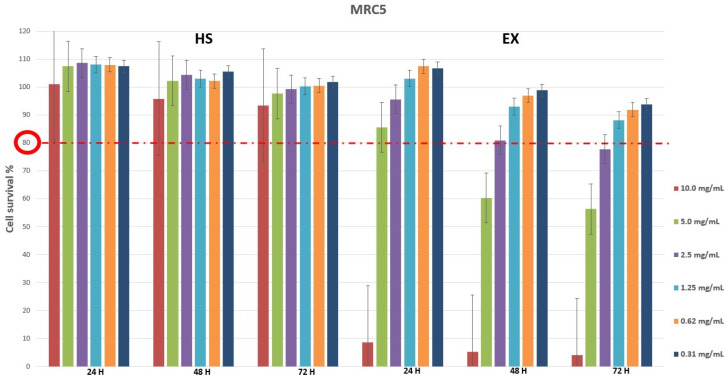
Cytotoxicity evaluation of HS and EX in MRC5 cells after 24, 48, and 72 h treatment by performing classic MTT assay. The data represent the mean ± standard deviation (SD) of three independent experiments. The graph showed high survival of MRC5 cells at all doses tested except for 10.0 mg/mL and 5.0 mg/mL of EX at 48 h and 72 h.

**Figure 2:**
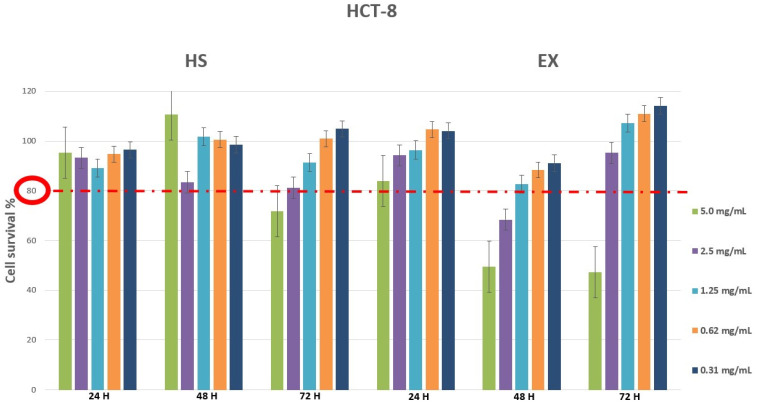
Cytotoxicity evaluation of HS and EX in HCT-8 cells after 24, 48, and 72 h treatment by performing classic MTT assay. The data represent the mean ± standard deviation (SD) of three independent experiments. The graph showed survival of HCT-8 cells above the reference threshold up to the concentration of 2.5 mg/mL at 72 h for both HS and EX.

Cytotoxicity evaluation of HS and EX was assessed (tested range 0.31–10.0 mg/mL) by performing MTT assay on MRC5 and HCT-8 cell lines at different exposure times (24, 48, 72 h). The results, shown in Figure 1 and Figure 2, demonstrated that HS solution guaranteed cell viability well beyond the chosen reference threshold (80%) at all times tested. Instead, high concentrations (10.0–5.0 mg/mL) of EX reduced cell viability, while the concentration of 2.5 mg/mL was well tolerated (Figure 1). Moreover, HCT-8 cells exposed to HS and EX showed high viability up to the concentration of 2.5 mg/mL up to 72 h (Figure 2).

**Figure 3:**
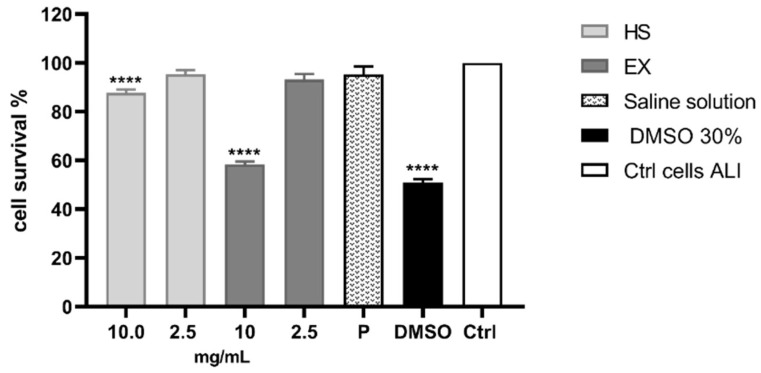
Cytotoxicity evaluation of HS and EX by performing MTT assay after ALI exposure. The graph showed the percentage of cell survival after ALI exposure to HS and EX at 10 mg/mL and 2.5 mg/mL. The data represent the mean ± standard deviation (SD) of three independent experiments; **** *p* < 0.001.

Cell viability was also evaluated by ALI exposure, which is the most physiologically relevant assay for bronchial epithelial cell lines. It was performed by exposing the cells to all fractions and components of aerosols generated by the selected substances [65]. As can be seen from the graph (Figure 3), the exposure method worked since the DMSO solution caused 50.97% of cell death, while the physiological solution did not show cytotoxic relevance under the experimental conditions. Therefore, it was possible to state that we carried out a high cell survival after exposure to HS and EX, both at 10 mg/mL (87.63% and 58.38%, respectively) and 2 mg/mL (95.32% and 95.15%, respectively).

Based on the results obtained on the MRC5 and HCT-8 cell lines, the antiviral activity of coronavirus strains was evaluated at the concentration range of 0.08–2.5 mg/mL. The exposure time was necessarily 72 h since HCoV-229E, and HCoV-OC43 strains begin to cause CPE from 3 days onwards after cell infection, with an average of 5 days. Finally, the MOI used was equal to 0.01.

**Figure 4:**
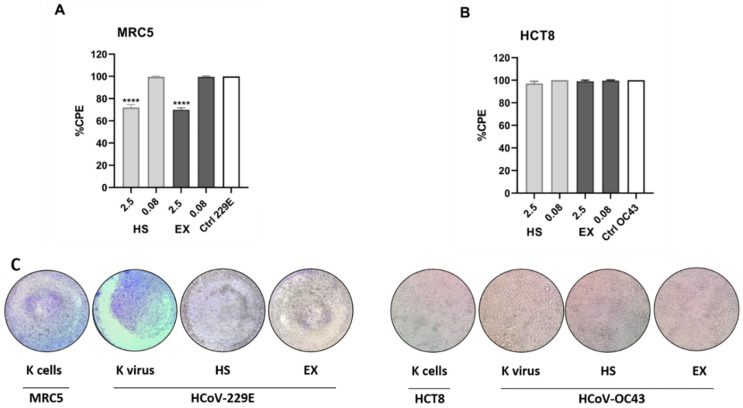
(**A**) Percentage of the CPE calculated by MTT assay of HCoV-229E strain on MRC5 cells after exposure to HS and EX substances. The graph shows the highest and lowest concentrations tested, respectively. The 2.5 mg/mL concentration contributed to a 30% reduction in CPE. In contrast, the concentration equal to 0.08 mg/mL had no antiviral activity showing a cytopathic effect equal to the K virus. (**B**) Percentage of the CPE calculated by MTT assay of HCoV-OC43 strain on HCT-8 cells after exposure to HS and EX substances. The graph shows the highest and lowest concentrations tested, respectively. None of the tested doses showed antiviral activity showing a cytopathic effect equal to the K virus. (**C**) Morphological change and CPE due to HCoV-229E (left) and HCoV-OC43 strains (right) read-out 72 h post administration (2.5 mg/mL) by ALI exposure. MOI 0.01; All data were calculated as the percentage of the CPE with respect to the negative control (K cells). The data represent the mean ± standard deviation (SD) of three independent experiments; **** *p* < 0.001.

Figure 4A shows the CPE results of the HCoV-229E strain on MRC5 cells after exposure to HS and EX. A reduction in viral CPE (30%) was evident only at the highest concentration tested, i.e., 2.5 mg/mL, with a CPE equal to 70% compared to the K virus (Figure 4C, left). Regarding results concerning the HCoV-OC43 strain on HCT-8 cells, no antiviral activity was evident at the concentrations tested (Figure 4B,C right).

HCoV-229E binds to mammalian cells through its spike’s receptor-binding domain (RBD) [14].

Molecular modeling experiments were performed to confirm the interactions of the two studied molecules at the interface between the spike protein of 229E and APN.

Different regions of the spike proteins were studied, as suggested by the literature. Notably, for 229E and APN, a recent paper [73] suggested a study at the interface between spike and APN. In this case, inhibition of the interaction spike/APN is expected, and our study confirmed this interaction with inhibition activity. The other region studied for the spike protein of 229E was suggested by a different study [84] where a possible cofactor activity was studied. If the studied HS and EX could act as cofactors, the global activity would create an equilibrium between the inhibition/cofactor activity for the single molecule. As already reported for similar oligosaccharides [84], the presence of the molecule on the surface (but not at the interface between the two proteins; they have to interact in order for the molecule to act as a cofactor) of the spike protein of SARS-CoV-2 revealed a significant conformational adjustment of the spike RBD, resulting in increased contact with the ACE2 protein and, hence, a cofactor activity.

**Figure 5:**
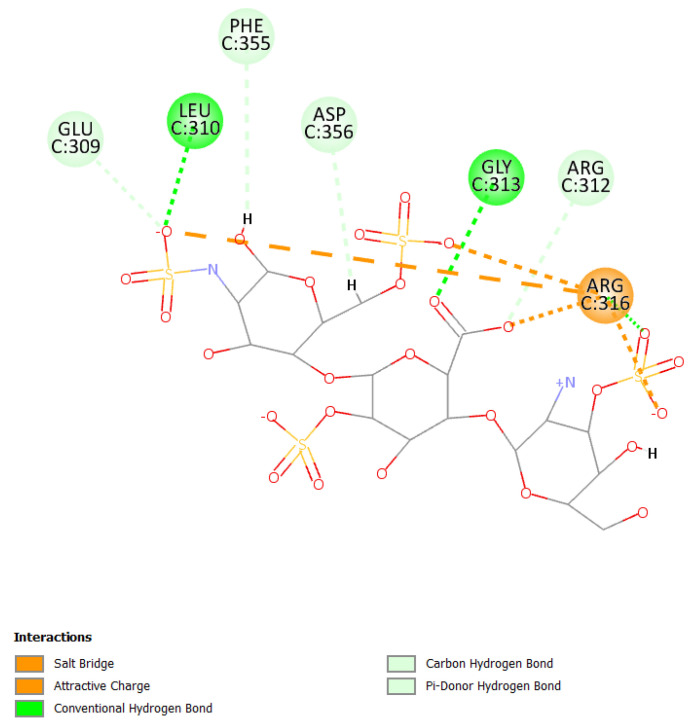
2D pose of heparan at the interface between the crystal structures of HCoV-229E RBD Class V and with human APN (PDB ID: 6U7G).

**Figure 6:**
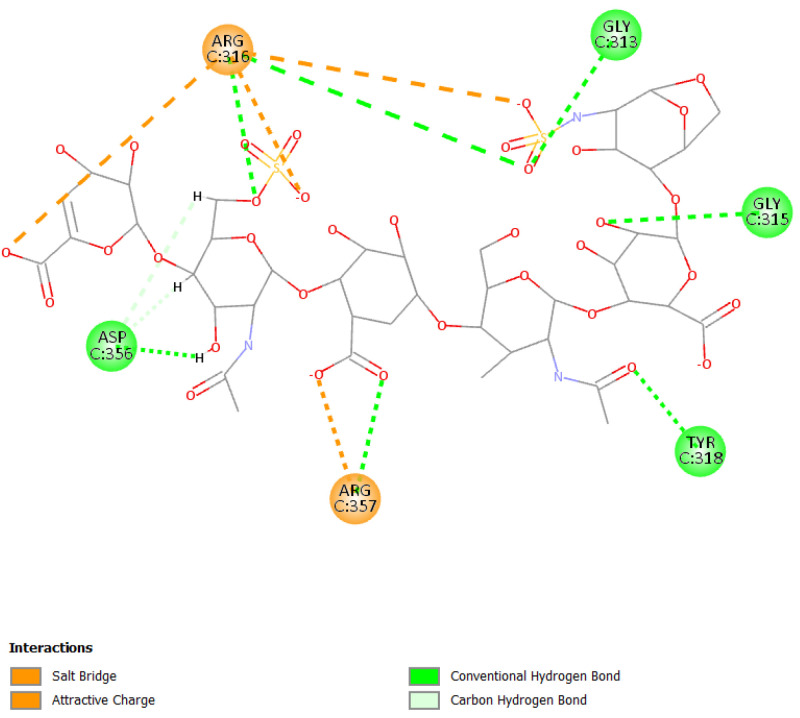
2D pose of enoxaparin at the interface between the crystal structures of HCoV-229E RBD Class V and with human APN (PDB ID: 6U7G).

Mainly, molecular docking experiments and molecular dynamics were performed. From the 2D poses shown in Figure 5 and Figure 6, it can be seen that both compounds interact with residues at the interface between the two subunits with Arg357 and Arg316, as already reported [73].

Primarily, HS can establish a salt bridge and electrostatic interactions with the residue Arg316 due to sulfate groups and can also form hydrogen bonds with Leu310 and Gly313 (Figure 5).

Otherwise, the binding pose of EX is influenced by the different sulfate and carboxyl groups present in the molecule. The presence of the two functional groups allows the formation of a salt bridge and electrostatic interactions with Arg316 and Arg357; moreover, the molecule can form several hydrogen bonds with other residues such as Asp356, Tyr318, Gly313, and Gly315 (Figure 6).

**Figure 7:**
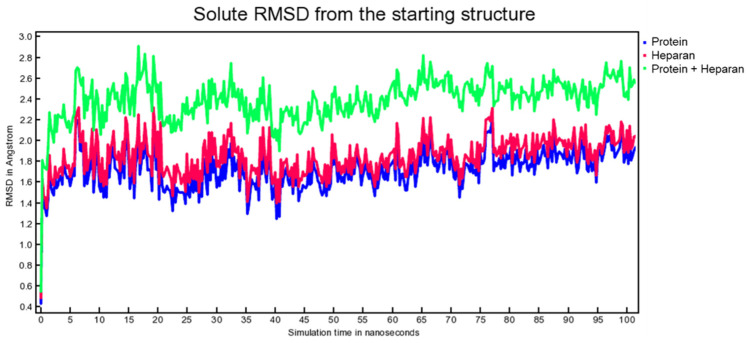
RMSD from the starting structure as a function of simulation time for the spike-HS complex.

**Figure 8:**
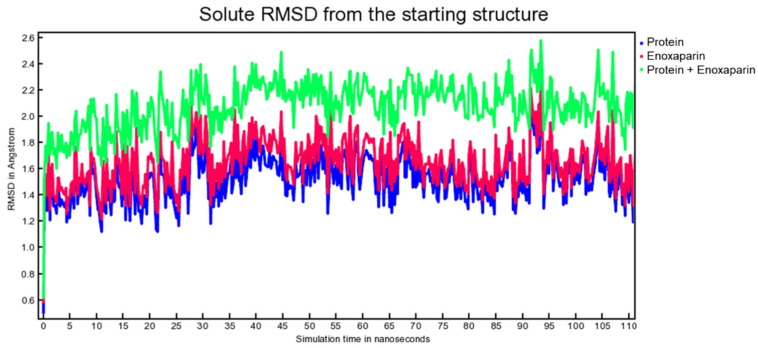
RMSD from the starting structure as a function of simulation time for the spike-EX complex.

Molecular dynamic experiments were performed to study the formed complexes over time. For both experiments, the docked conformation was used as a starting geometry, and 100 ns of dynamic were conducted in water as described in the experimental section. Generally, both the studied complexes revealed stability over time as measured by the slight fluctuation of the root-mean-square deviation (RMSD) of atomic positions reported in Figure 7 for the spike-HS complex and Figure 8 for the spike-EX complex.

**Figure 9:**
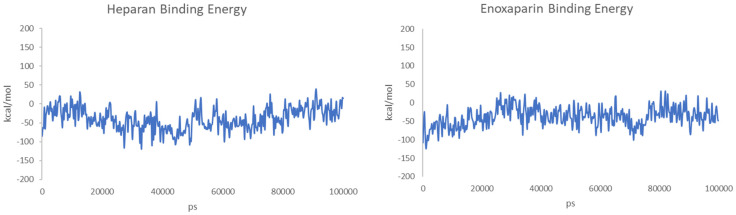
Calculated binding energies of HS (**left**) and EX (**right**) to the surface of spike protein over 100 ns of MD.

The binding energy was also calculated for each snapshot of the MD simulations, and the values are reported in Figure 9.

For HS, the starting contact residues (Figure S3) from the docking calculation with Leu310 and Gly313 are disengaging/engaging over time (Figures S4–S6), but the electrostatic interactions with the residue Arg316 of the sulfate groups are present over time of the MD, and a 100 ns the Arg321 is taking over. Other residues are also engaging interactions, particularly, Arg357, Arg357, Arg312, and Gly315, Gly313 (at 27 ns, Figure S4).

EX has a similar behavior: losing some initial contact (Figure S7) and gaining some different, but pivoting around the Arg316 and Arg357, constantly interacting with the molecule. Notably, at 26 ns (Figure S8), the molecule is interacting with Arg357; at 77 ns (Figure S9) is interacting with Arg321, Arg311, Arg357, and Asn319 and still interacting with Arg316. At 100 ns, the molecule interacts with Arg316, Asn319, and Tyr318 (Figure S10).

Moreover, the activity as a possible cofactor for the viral entry process of the HCoV-229E was also considered and modeled to verify a possible competition of the inhibitor activity for both molecules. Indeed, the entry process of the SARS-CoV-2 virus requires a combination of HS and heparin on the cell surface to act as a cofactor and mediate the interaction between the spike protein and ACE [84]. If the studied HS and EX were able to act as cofactors, the global activity would result in an equilibrium between the inhibition/cofactor activity for the single molecule. To verify this, the molecules were first docked to the surface of the spike protein interacting with APN. Then, an MD simulation was performed to analyze the binding activity over time.

**Figure 10:**
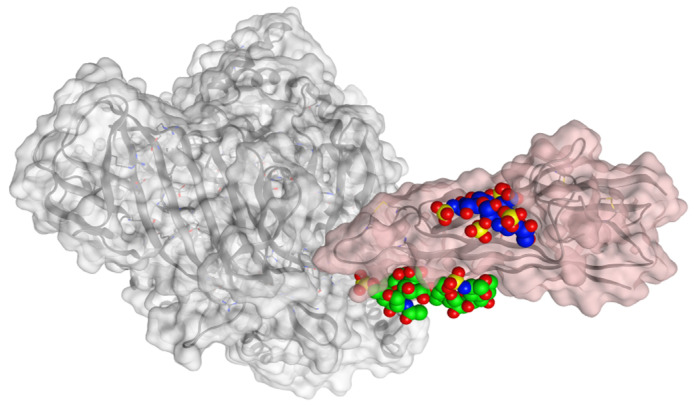
Calculated binding poses with spike protein (pink) of HS (blue) and EX (green). APN protein is shown in grey, at the surface of the crystal structures of HCoV-229E RBD Class V (PDB ID: 6U7G).

The calculated binding poses between the spike protein and HS/EX are reported in Figure 10.

**Figure 11:**
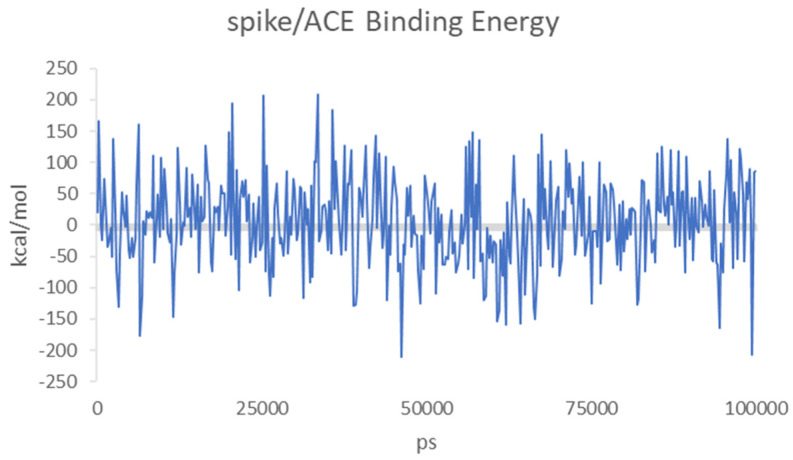
Calculated binding energies of spike/APN for HCoV-229E over 100 ns of MD.

**Figure 12:**
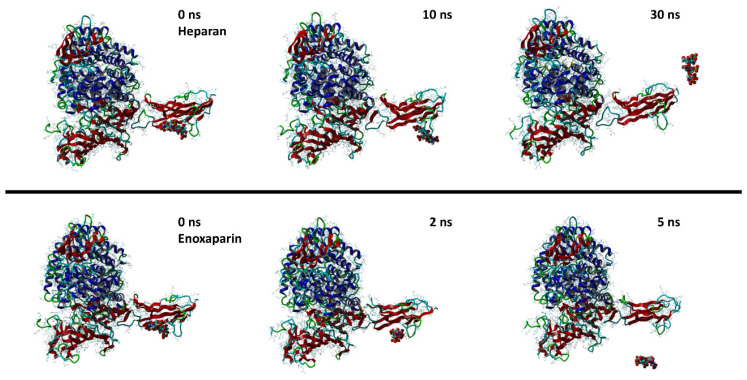
Snapshots from MD simulations of HS (**up**) and EX (**down**) with the surface of spike/APN complex.

Both molecules can interact in two close but different binding sites on the surface of the spike protein when in complex with the APN protein. For HS, the main interactions are with Lys388 (H-acceptor and ionic), Thr412 (H-acceptor), Val325 (H-acceptor), Ile327 (H-acceptor), Glu334 (H-acceptor), and Leu329 (H-acceptor). EX differently interacts with: Asn307 (H-acceptor), Asn326 (H-acceptor), Leu210 (H-acceptor), Glu309 (H-acceptor), and Arg316 (H-acceptor and ionic interactions). Once the capabilities of the surface interaction are verified, the question to answer is if this can be maintained over time and if this interaction could increase the interaction between APN and spike. As already reported for similar oligosaccharides, the presence of the molecule on the surface of the spike protein of SARS-CoV-2 revealed a significant conformational adjustment of the spike RBD, resulting in increased contact with the ACE2 protein and, hence, a cofactor activity [84]. MD experiments were settled to verify or discard this assumption for spike–APN interaction of HCoV-229E. A total of 100 ns of dynamic were performed for the two complexes HS/spike/APN and EX/spike/APN. A third dynamic was performed to calculate the binding interaction over time between the spike and APN themselves. As reported in Figure 11, the calculated binding energy was constant over time between the spike and the APN. The increase of the interaction energy between spike and APN was not revealed from the HS/spike/APN and EX/spike/APN experiments. As reported in Figure 12, both molecules could not maintain their surface binding poses over time; hence, no difference in the binding energies between APN and spike was revealed when HS and EX were present. Mainly, HS started to lose contact after 10 ns, and at 30 ns it is wholly disjoined from the spike surface; Otherwise, after only 2 ns, EX is no longer at the binding site, and at 5 ns, the molecule is entirely far away.

In conclusion, between the two postulated inhibition and cofactor mechanisms, the only one identified as possible by our calculation is the inhibition one for both molecules that well explain the activity measured in our reported in vitro experiments for HCoV-229E.

To study the activity of HS and EX in HCoV-OC43, attention was focused on a different protein. 9-*O*-acetylated sialic acids (9-*O*-Ac-Sias) were identified as the main ligand of the NTDs of lineage A β-coronaviruses as shown for the prototype BCoV virus and related human virus HCoV-OC43 [13]. Docking calculations were performed to identify a possible interaction between HS, EX, and the sialoglycan-binding site. This site is conserved in all other coronaviruses known to attach to 9-*O*-Ac-Sia-decorated oligosaccharides present at the surface of host cells targeted by these viruses. Occupying the sialoglycan-binding with high affinity will inhibit the virus recognition of 9-*O*-Ac-Sia-decorated oligosaccharides and inhibit cellular entry. To verify this assumption, docking calculations were performed using the HCoV-OC43 spike (PDB ID: 6NZK), and the binding results were compared to the original ligand (methyl 9-*O*-acetyl-5-acetamido-3,5-dideoxy-d-glycero-α-d-galacto-non-2-ulopyranosidonic acid).

As expected from the experimental results concerning the OC43 strain on HCT-8 cells where no antiviral activity was evident, the docking calculation suggested that no relevant interactions are present between HS/EX with the OC43 spike sialoglycan-binding site. According to our docking calculation, HCoV-OC43 was not inhibited by heparan sulfate and enoxaparin because of the relatively lower free energies of binding of the two molecules compared with the original ligand pyranosidonic acid. In fact, the calculated energy of binding of the original pyranosidonic acid ligand was −6.40 kcal/mol, whereas the energies of binding of HS and EX were −6.28 and −5.77 kcal/mol, respectively. A more negative free energy of binding would result in better interaction. Heparan sulfate and enoxaparin have not achieved the same value (−6.40 kcal/mol) of the original ligand; hence, they are unsuitable for the same binding site.

This would result in preferential binding to the 9-*O*-Ac-Sia-decorated oligosaccharides at the cellular level of the sialoglycan-binding site spike protein and, hence, a non-relevant competition/inhibition of this activity by the two studied molecules as demonstrated in our in vitro experiments.

## 4. Conclusions

HS solution guaranteed cell viability well beyond the chosen reference threshold (80%) at all times tested. Instead, high concentrations (10.0–5.0 mg/mL) of EX reduced cell viability, while the concentration of 2.5 mg/mL was well tolerated. Moreover, EX and HS showed cytotoxicity in a dose-dependent fashion in ALI exposure. Both EX and HS showed a slight viral 229E inhibition. In fact, a reduction in virus CPE of about 30% was observed with the tested dose equal to 2.5 mg/mL against HCoV-229E. All other doses tested showed no inhibition, and no inhibition was observed for the HCoV-OC43 strain at any of the doses tested.

HS increases the interaction with the cellular receptor or, in itself, can represent an alternative receptor for the entry of the virus into cells [85,86]. It is now well established that the binding of a virus to its cellular receptor can be prevented by the presence of a synthetic receptor acting as a competitor [87,88]. However, while for HIV this aspect has been confirmed [89], in the case of the coronavirus, this type of action for glycans and proteoglycans has yet to be fully confirmed/clarified [90].

In our study, we have shown that the presence of HS or EX can interfere by reducing the effects of the virus. Furthermore, this antiviral activity of HS and EX was found only against an α-coronavirus. Most likely, as suggested by the docking study, the presence of the two molecules reduces the viral load but not completely inhibiting it since it would act as a partial inhibitor of the link with APN [91]. On the contrary, no inhibitory activity could be hypothesized on β-coronavirus HCoV-OC43. Our results open a new probable mechanism of action of proteoglycans as selective antiviral competitive inhibitors.

## Data Availability

Not applicable.

## Supplementary Materials

The following supporting information can be downloaded at: https://www.mdpi.com/article/10.3390/v15030663/s1, Figures S1. ALI exposure system; Figure S2. Perspex aerosol exposure chamber; Figures S3–S10 Heparan and Enoxaparin MD snapshots and calculated 2D interactions [92].
