# Supplementary material for: Heparan Sulfate and Enoxaparin Interact at the Interface of the Spike Protein of HCoV-229E but Not with HCoV-OC43
Source: Viruses. 2023 Mar 1;15(3):663. doi: 10.3390/v15030663 (PMC10056857; doi:10.3390/v15030663)
Supplement: Supplementary file 1 [file viruses-15-00663-s001.zip › viruses-2003415-supplementary.pdf]

viruses

Supplementary Materials

Heparan Sulfate and Enoxaparin Interact at the Interface of the Spike Protein of HCoV-229E but Not with HCoV-OC43

Virginia Fuochi [1,2,†], Giuseppe Floresta [3,†], Rosalia Emma [1], Vincenzo Patamia [3], Massimo Caruso [1,2], Chiara Zagni [3], Federica Ronchi [4], Celestino Ronchi [4], Filippo Drago [1], Antonio Rescifina [3,*] and Pio Maria Furneri [1,2,*]

1 Department of Biomedical and Biotechnological Sciences (Biometec), University of Catania, 95124 Catania, Italy
2 Center of Excellence for the Acceleration of Harm Reduction (Coehar), University of Catania, 95124 Catania, Italy
3 Department of Drug and Health Sciences (DSFS), University of Catania, 95125 Catania, Italy
4 Delim Cosmetics & Pharma S.R.L., 20090 Vimodrone, MI, Italy
* Correspondence: arescifina@unict.it (A.R.); furneri@unict.it (P.M.F.)
† These authors contributed equally to this work.

Table of content

Figure S1. ALI exposure system. S2
Figure S2. Perspex aerosol exposure chamber. S2
Figure S3. Heparan snapshot at 0 ns (up) and calculated 2D interactions. S3
Figure S4. Heparan snapshot at 27 ns (up) and calculated 2D interactions. S4
Figure S5. Heparan snapshot at 77 ns (up) and calculated 2D interactions. S5
Figure S6. Heparan snapshot at 100 ns (up) and calculated 2D interactions. S6
Figure S7. Enoxaparin snapshot at 0 ns (up) and calculated 2D interactions. S7
Figure S8. Enoxaparin snapshot at 26.75 ns (up) and calculated 2D interactions. S8
Figure S9. Enoxaparin snapshot at 71 ns (up) and calculated 2D interactions. S9
Figure S10. Enoxaparin snapshot at 100 ns (up) and calculated 2D interactions. S10

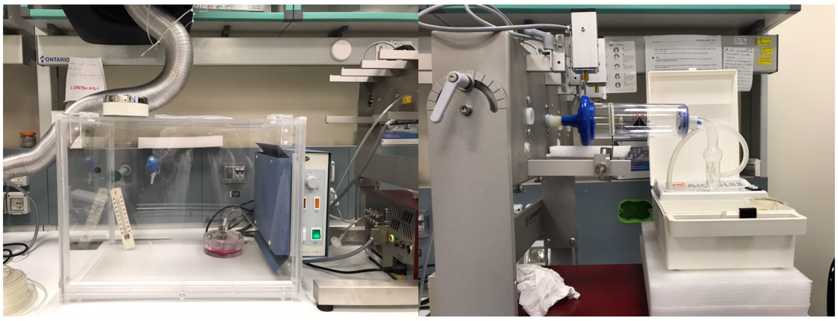

Figure S1. ALI exposure system: Thermostatic incubator where cells were maintained during the exposure (left). Nebulizer (Microlux Medel) attached to Borgwaldt LM4E vaping machine to vaporize solutions (right). Photos were edited using the GIMP image manipulation program (version 2.10.14).

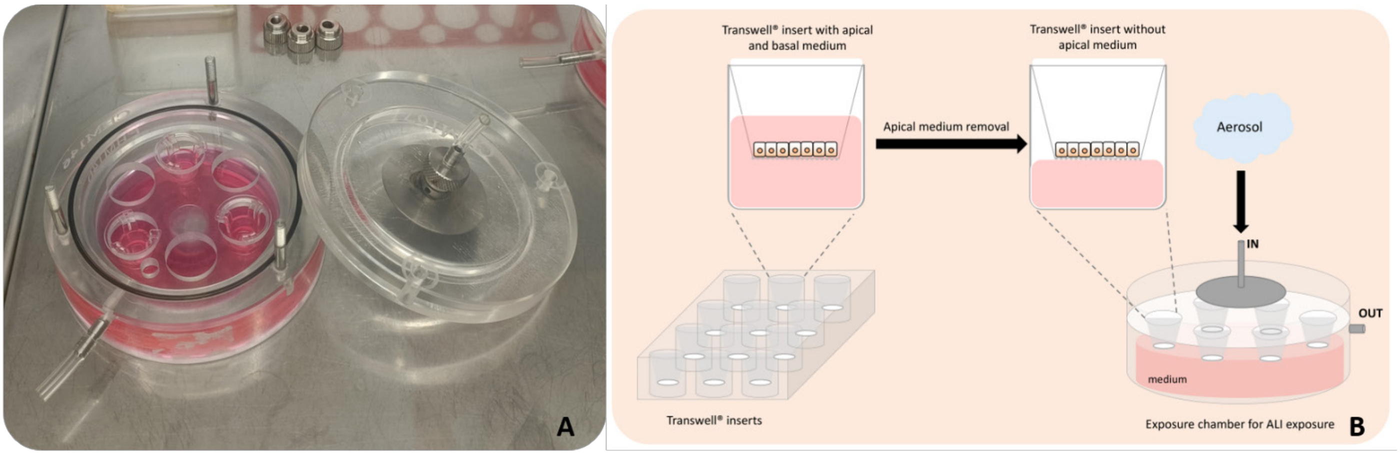

Figure S2. A) Perspex aerosol exposure chamber. B) The exposure chamber introduces the test aerosol through a single gas inlet. A symmetrical aerosol distribution disc ensures uniform cellular air-liquid interface (ALI) exposure avoiding the accumulation of aerosol inside the system. Specifically, cells cultured in Transwell inserts were deprived of the apical medium, and then placed in the exposure chamber contained the medium at the basal compartment. The exposure chamber is connected to the LM4 vaping machine to deliver the test aerosol [1].

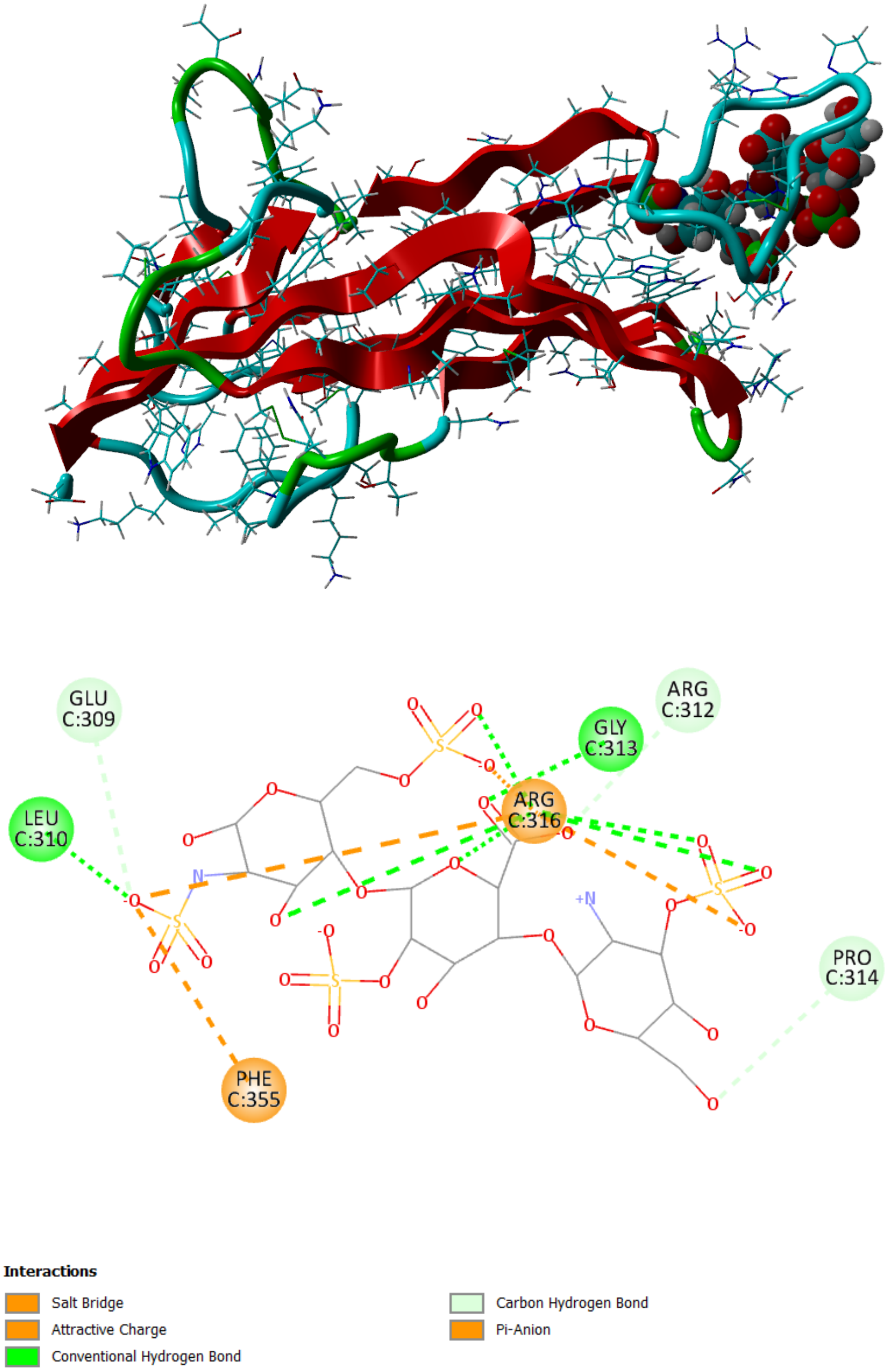

Figure S3. Heparan snapshot at 0 ns (up) and calculated 2D interactions in HCoV-229E in its spike's receptor-binding domain (RBD) (PDB ID: 6U7G).

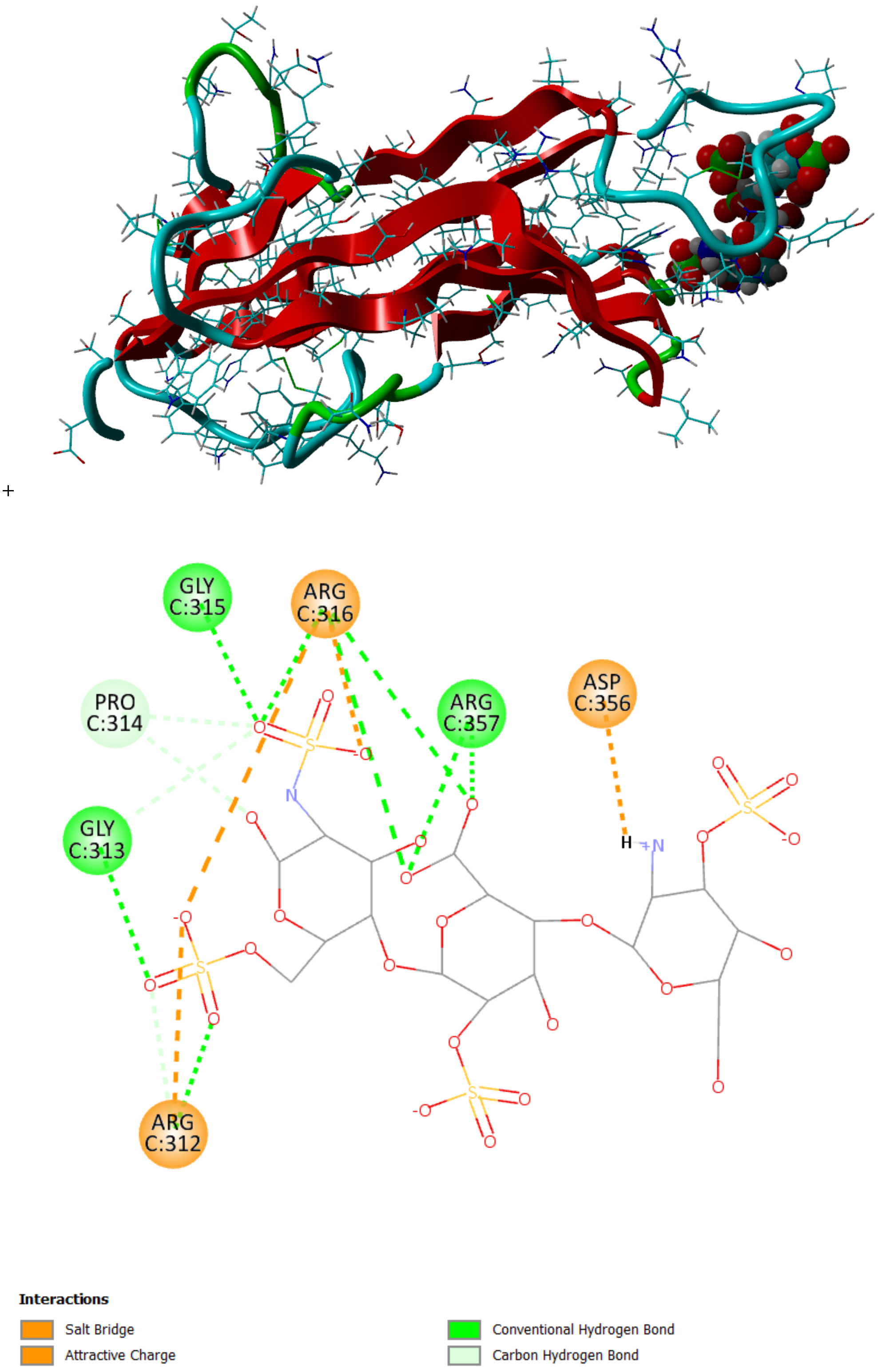

Figure S4. Heparan snapshot at 27 ns (up) and calculated 2D interactions in HCoV-229E in its spike's receptor-binding domain (RBD) (PDB ID: 6U7G).

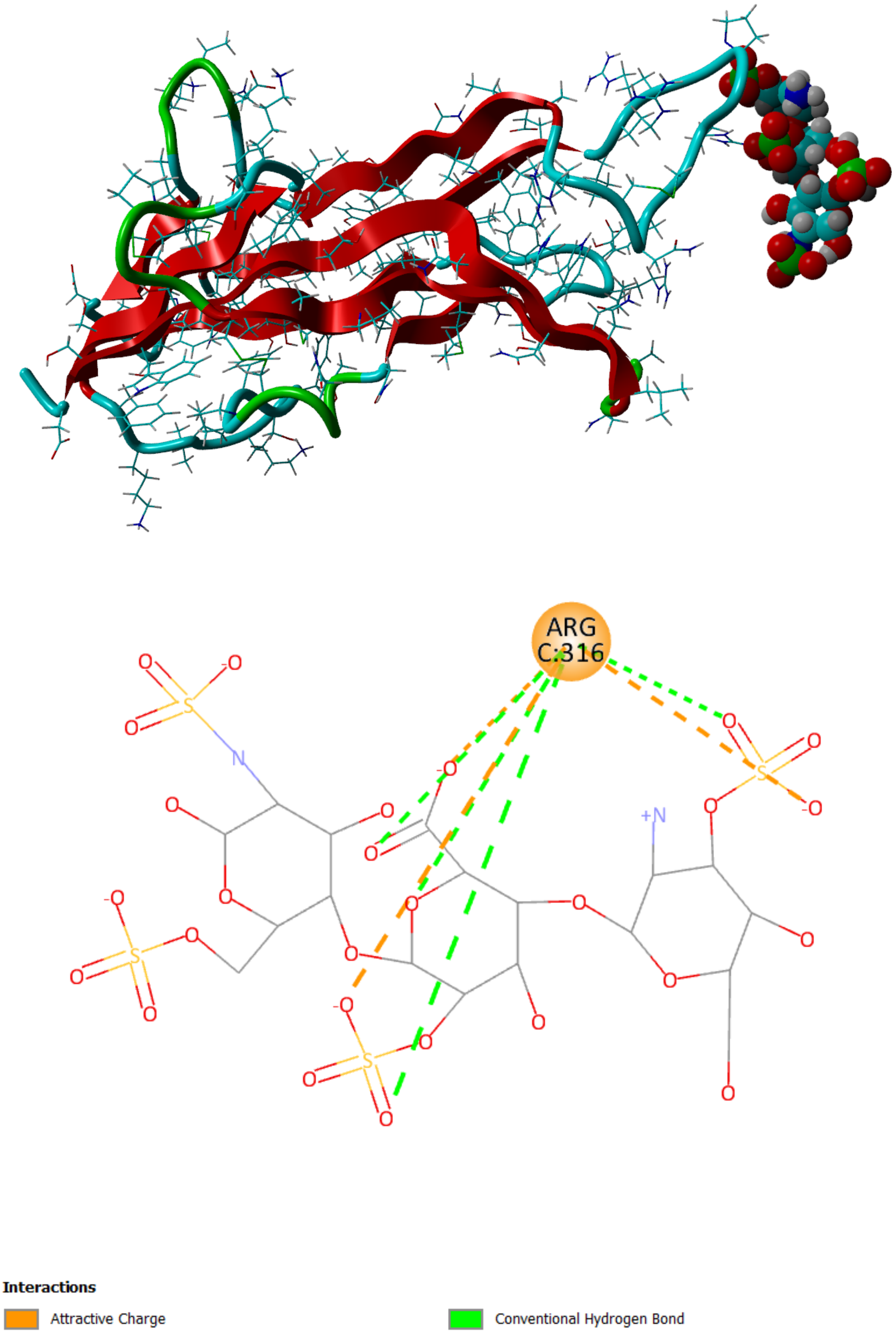

Figure S5. Heparan snapshot at 77 ns (up) and calculated 2D interactions in HCoV-229E in its spike's receptor-binding domain (RBD) (PDB ID: 6U7G).

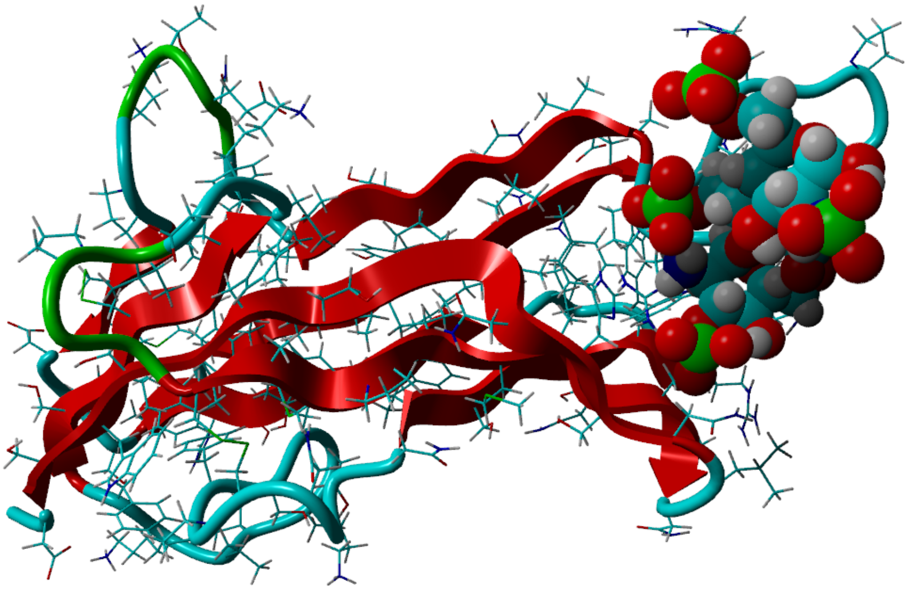

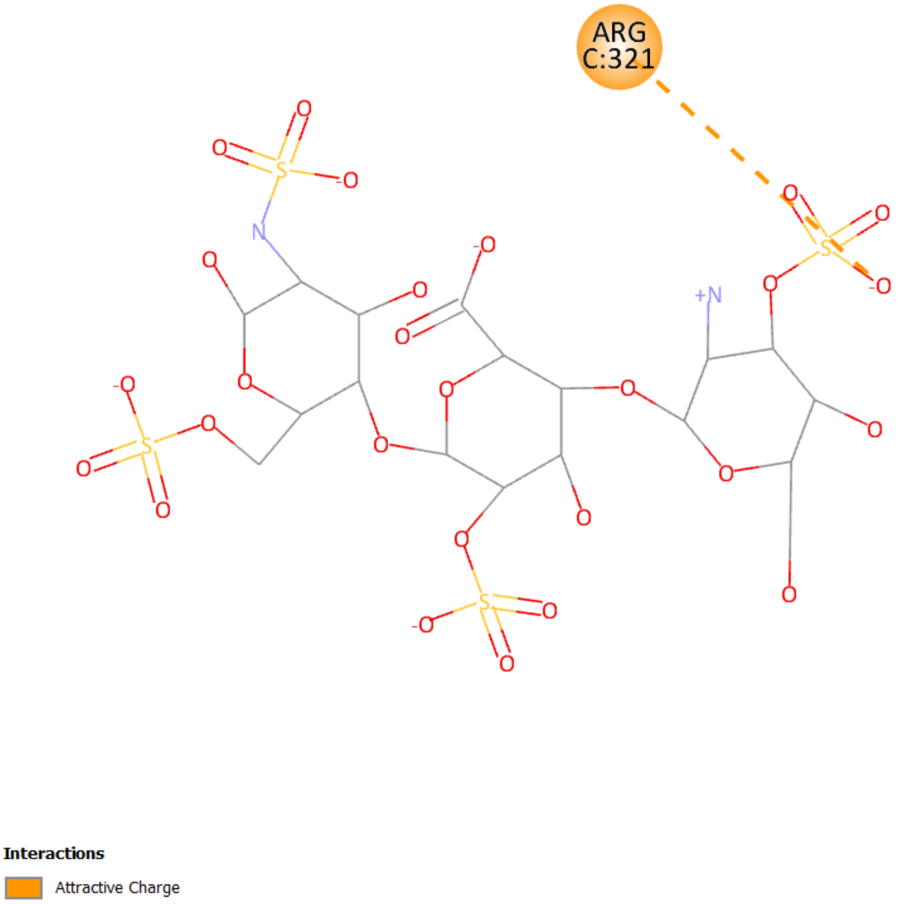

Figure S6. Heparan snapshot at 100 ns (up) and calculated 2D interactions in HCoV-229E in its spike's receptor-binding domain (RBD) (PDB ID: 6U7G).

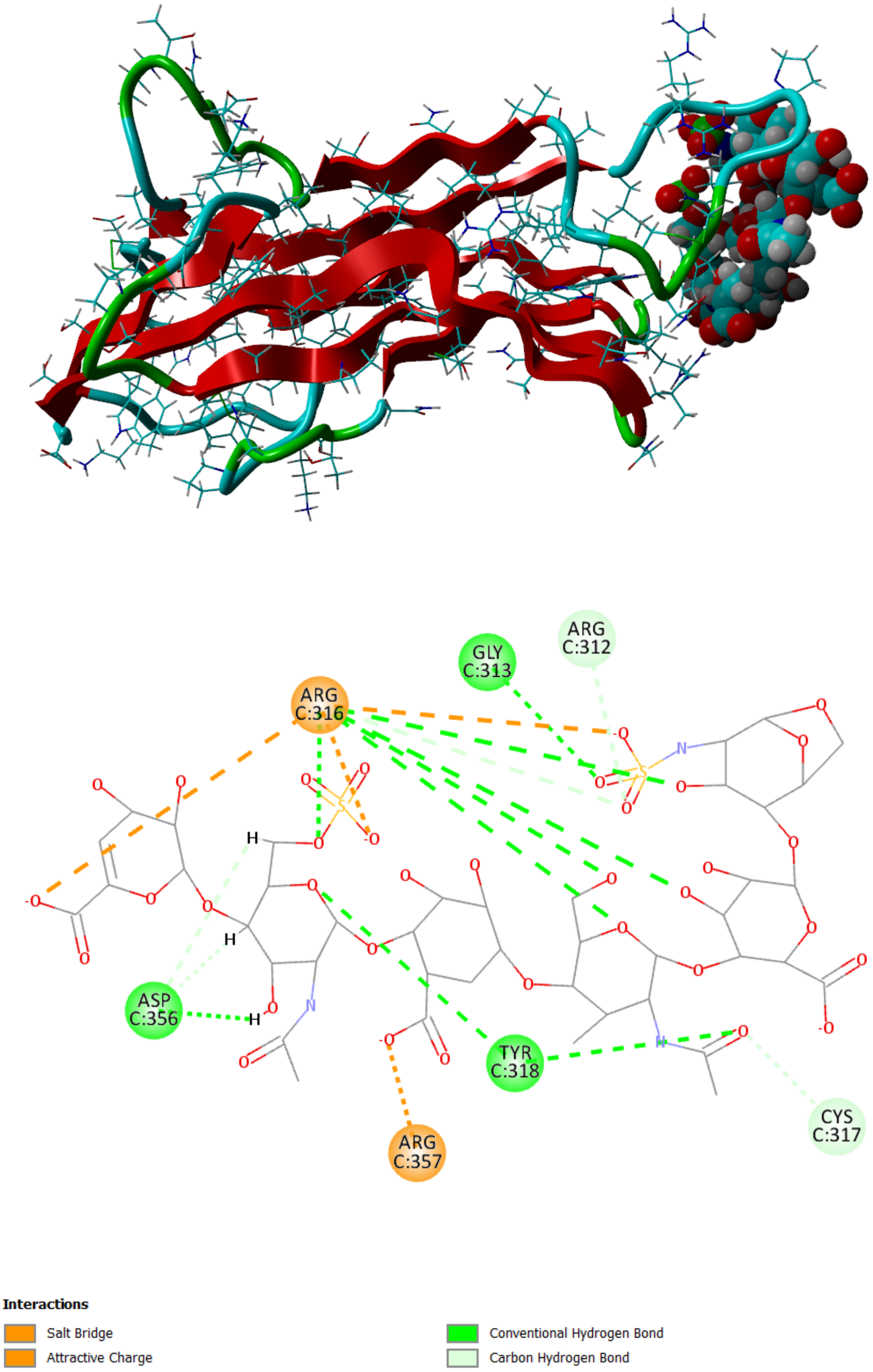

Figure S7. Enoxaparin snapshot at 0 ns (up) and calculated 2D interactions in HCoV-229E in its spike's receptor-binding domain (RBD) (PDB ID: 6U7G).

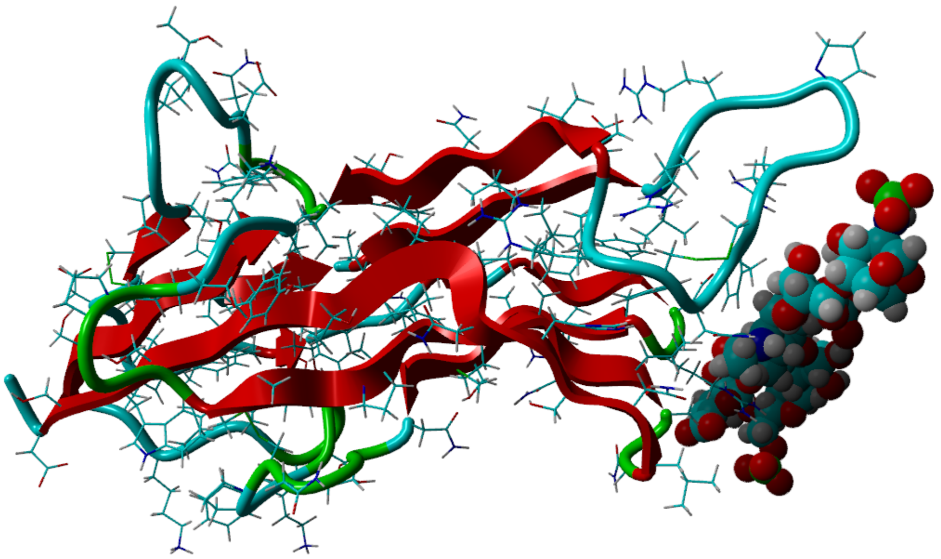

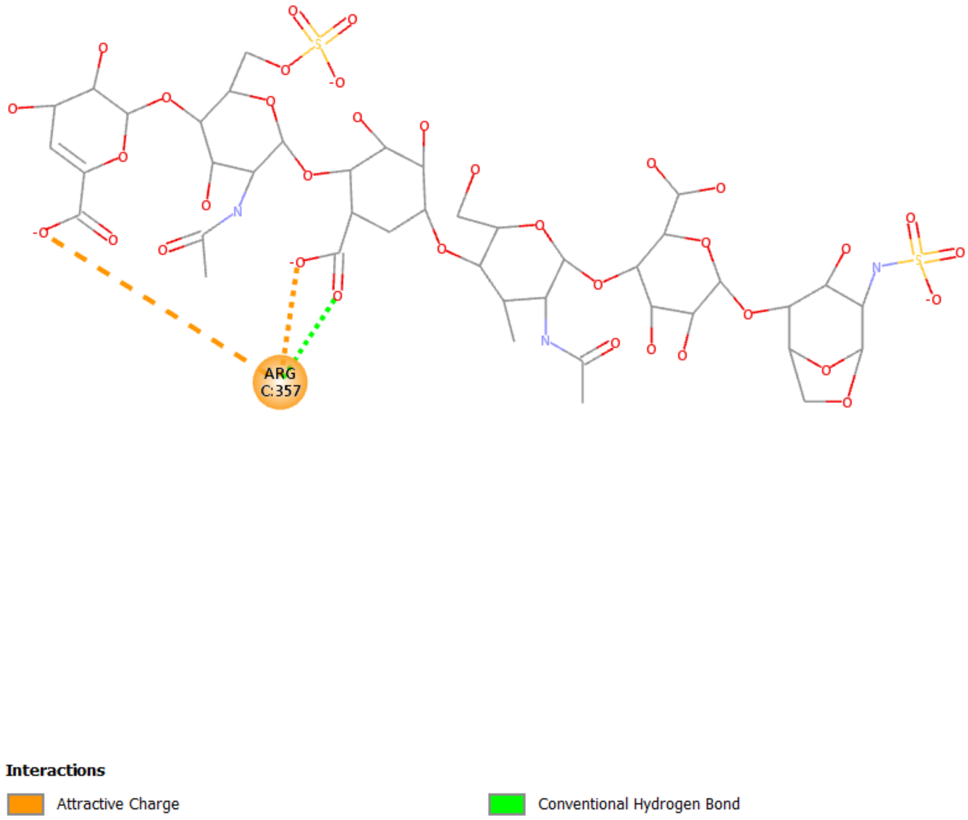

Figure S8. Enoxaparin snapshot at 26.75 ns (up) and calculated 2D interactions in HCoV-229E in its spike's receptor-binding domain (RBD) (PDB ID: 6U7G).

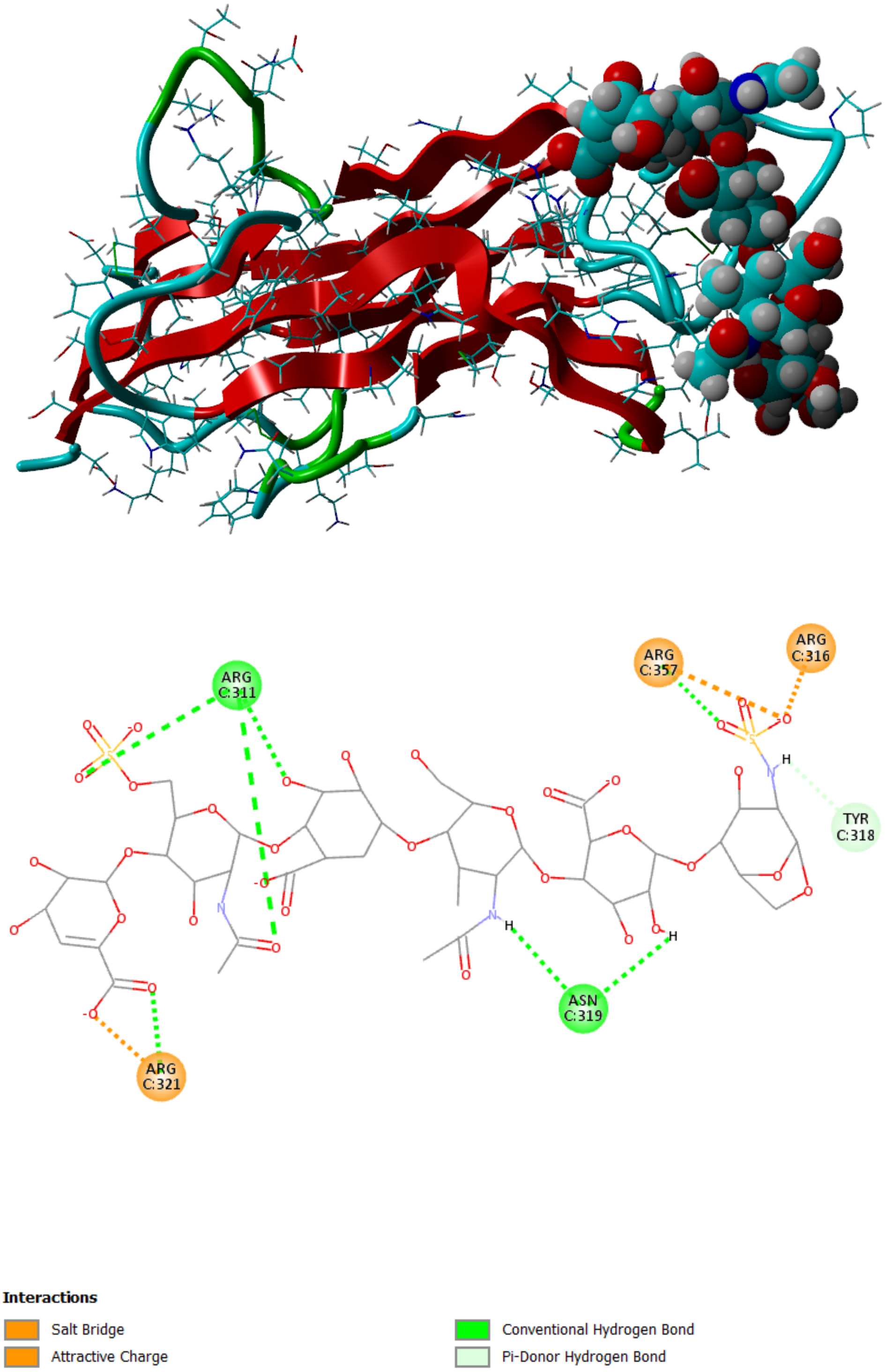

Figure S9. Enoxaparin snapshot at 71 ns (up) and calculated 2D interactions in HCoV-229E in its spike's receptor-binding domain (RBD) (PDB ID: 6U7G).

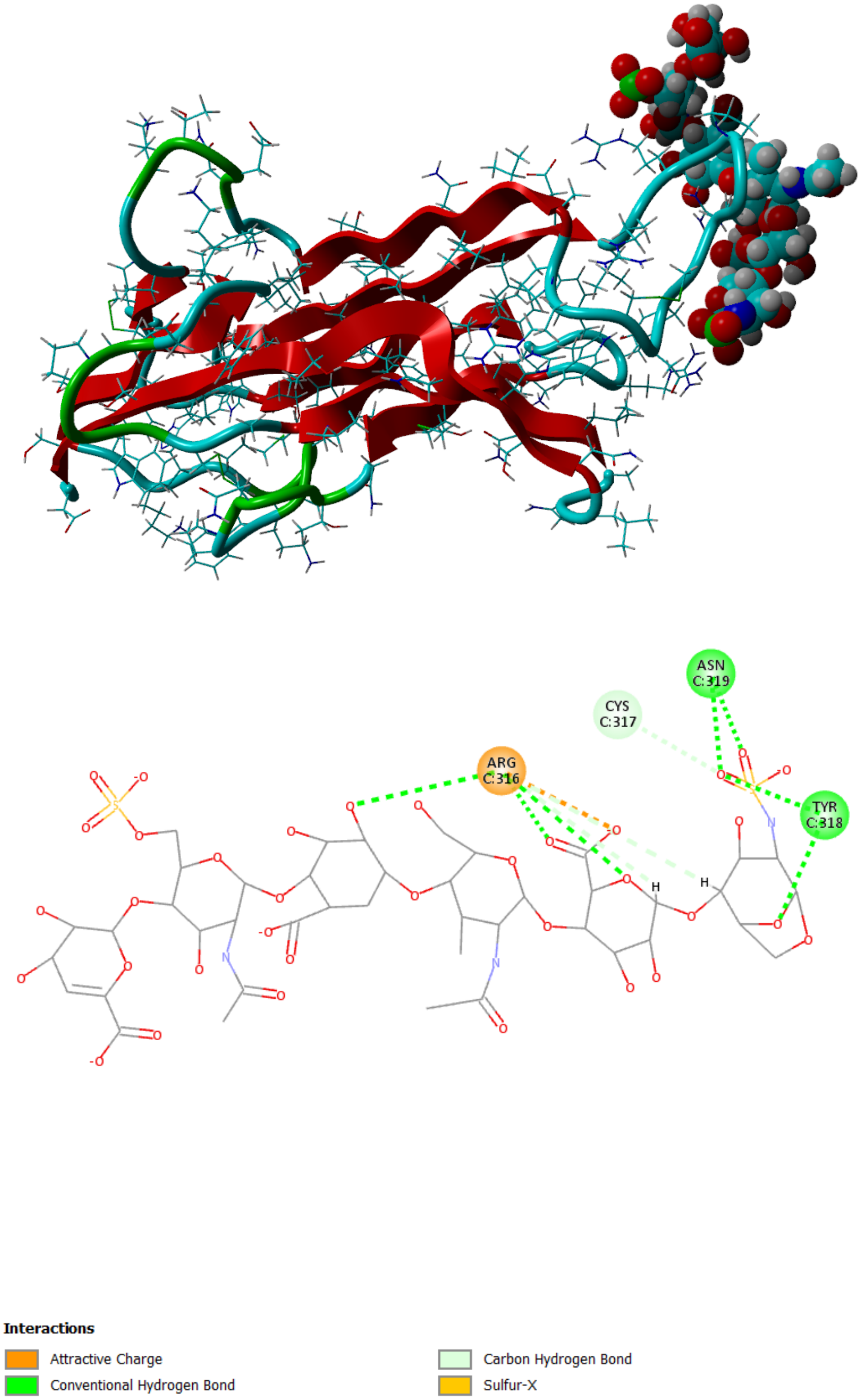

Figure S10. Enoxaparin snapshot at 100 ns (up) and calculated 2D interactions in HCoV-229E in its spike's receptor-binding domain (RBD) (PDB ID: 6U7G).

Reference

1. Caruso, M.; Distefano, A.; Emma, R.; Di Rosa, M.; Carota, G.; Rust, S.; Polosa, R.; Zuccarello, P.; Ferrante, M.; Raciti, G.; Li Volti, G., Role of Cigarette Smoke on Angiotensin-Converting Enzyme-2 Protein Membrane Expression in Bronchial Epithelial Cells Using an Air-Liquid Interface Model. *Front. Pharmacol.* **2021**, 12, 652102.
